# Estimating the Responses of Hydrological and Sedimental Processes to Future Climate Change in Watersheds with Different Landscapes in the Yellow River Basin, China

**DOI:** 10.3390/ijerph16204054

**Published:** 2019-10-22

**Authors:** Xue Li, Jian Sha, Yue Zhao, Zhong-Liang Wang

**Affiliations:** 1 Tianjin Key Laboratory of Water Resources and Environment, Tianjin Normal University, Tianjin 300387, China; xli@tjnu.edu.cn (X.L.); shajian@tjnu.edu.cn (J.S.); 2 Water Environment Institute, Chinese Academy for Environmental Planning, Beijing 100012, China

**Keywords:** climate change, hydrological process, sediment, land use, Yellow River

## Abstract

This study concerned the sediment issue of the Yellow River basin. The responses of hydrological and sedimental processes to future climate change in two upland watersheds with different dominant landscapes were estimated. Four Representative Concentration Pathway (RCP) scenarios with different radiative forcing levels were considered. The outputs of eleven Global Climate Models (GCMs) were used to represent the future climate status of the 2050s and 2070s, and an ensemble means was achieved to avoid uncertainty. The Long Ashton Research Station Weather Generator (LARS-WG) was employed to downscale the outputs of GCMs for future site-scale daily weather data estimations. The Generalized Watershed Loading Functions (GWLF) model was employed to model the streamflow and sediment yields under various scenarios and periods. The results showed that there would be generally hotter and wetter weather conditions in the future. Increased erosion and sediment yields could be found in the study area, with lesser increments in sediment in woodland than in cultivated field. The peak of sediment would appear in the 2050s, and integrated measures for sediment control should be implemented to reduce erosion and block delivery. The multi-model approach proposed in this study had reliable performance and could be applied in other similar areas with modest data conditions.

## 1. Introduction

The sediment substance in water bodies is of great importance for the aquatic ecosystem [1,2]. Soil erosion and subsequent sediment transport is the main source of suspended sediment in riverine systems [3,4]. Among them, soil erosion mainly depends on the basic conditions such as the amount and intensity of precipitation as well as the land use cover of the watershed. The sediment transport is driven by watershed hydrological processes, which are determined by the watershed land use cover and weather conditions for both precipitation and temperature. Temperature and precipitation changes in future climate change are expected to greatly affect the available water in the basin [5,6], so as to alter original soil loss and sediment delivery characteristics [7,8,9]. In addition, differences in underlying surface conditions in different watersheds would lead to high uncertainties in the extent and magnitude of these variations [10,11]. Many studies have evaluated the response of runoff and sediment loading to climate and land use/land cover change, based on historical data and/or various modeling tools [12,13,14]. However, different areas possibly have different aspects of response behavior, and it is necessary to carry out case studies in critical areas.

The Yellow River, as the second longest river in China and the sixth longest river system in the world, is one of the most sediment-laden rivers in the world [15,16]. A large amount of sediments are discharged into the middle stream of the Yellow River due to the substantial erosions taking place in the Chinese Loess Plateau [17,18]. Excessive sediment yields reveal significant environmental threats to riverbed routes and aquatic ecosystems, for which effective control measures are needed. The sediment load on the Loess Plateau has decreased in the past few decades due to various conservation projects, such as increasing vegetation coverage and terrace construction [19,20]. However, such profits are being challenged and may be offset by future climate change. The hotter and wetter climate status may lead to various possible changes in streamflow and sediment yields in different areas with different underlying surface conditions. Thus, quantitative estimations of hydrological and sedimentary processes in response to future climate change in watersheds with different landscapes in the Yellow River Basin are significant to local management for better mitigation strategies and more efficient management practices.

In this study, two typical upland watersheds with different land-use cover conditions in the Loess Plateau were considered. A series of scenario analyses were, respectively, achieved in a cultivated land and grass-dominant watershed and in a woodland-dominant watershed. The effects of climate changes on hydrological and sedimental processes in different watersheds were estimated and compared. The main purposes of this study are to estimate how the streamflow and sediment yields will change under different climate change scenarios in different future periods and whether these changes in streamflow and sediment will be different in watersheds with different land use cover in the Loess Plateau. In addition, an integrated model application with a modest data requirement was proposed and implemented in the two study areas to realize the quantitative estimations. The LARS-WG model was used for downscaling analyses of future weather data estimations, and the Generalized Watershed Loading Functions (GWLF) model was used for watershed streamflow and sediment estimations. Four Representative Concentration Pathway (RCP) scenarios with different radiative forcing levels proposed by the Intergovernmental Panel on Climate Change (IPCC) were used as future climate change scenarios, including one mitigation scenario of RCP 2.6, two medium stabilization scenarios of RCP4.5 and RCP6.0, and one extreme high emission scenario of RCP8.5. Eleven Global Climate Models (GCMs) outputs of two future periods (2050s and 2070s) were considered, and an ensemble means was achieved to avoid uncertainty, the results of which were further downscaled by the LARS-WG model to generate site and daily scale synthetic weather data for scenario analyses in GWLF. The results revealed a general increase in sediment yields under future changed climate conditions and a great advantage of woodland in resistance stability with less sediment increases. The woodland increasing strategies were expected, and various soil conservation measures should be implemented in cultivated land. The approach of integrated multi-model applications used in this study is effective and may also be applicable in other areas with similar conditions.

## 2. Materials and Methods

### 2.1. Study Area and Data Source

**Figure 1:**
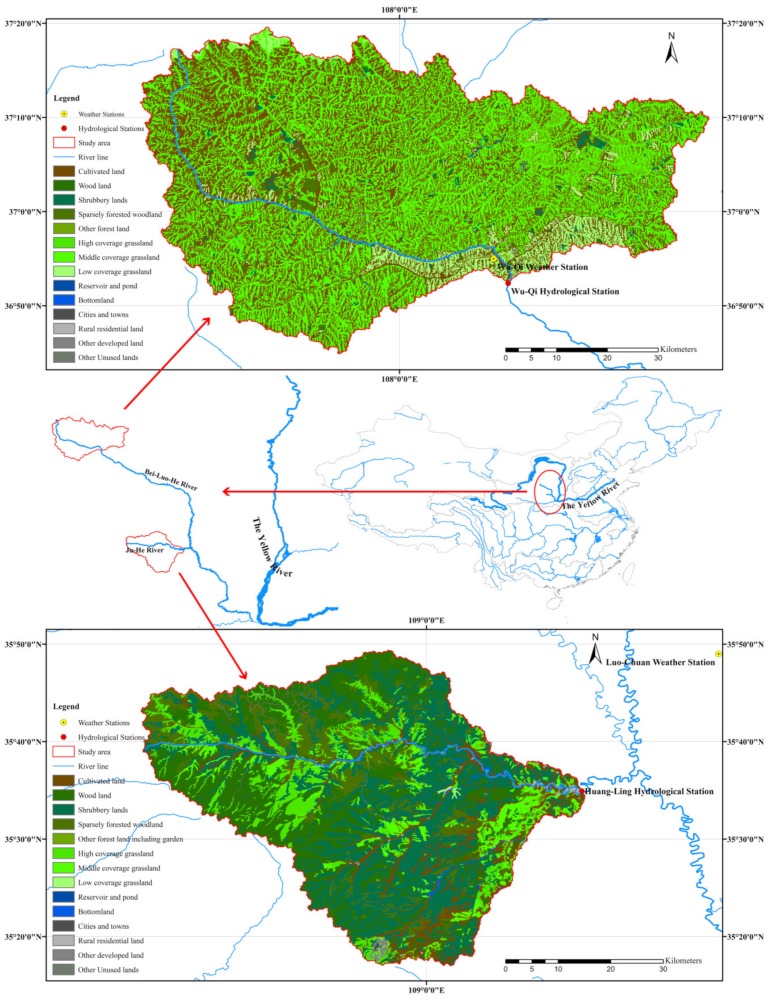
Geographical attributes of the study area.

This study was carried out in the Loess Plateau in the northeast of China, and two headwater watersheds of the Bei-Luo-He River were estimated (Figure 1). The Bei-Luo-He River extends from the south side of Bai-Yu-Shan Mountain in Shaanxi Province and drains into the Wei-He River, which is one of the main tributaries of the Yellow River, China. The source watershed of the Bei-Luo-He River located above Wu-Qi Hydrological Gauge Station was used as one of the study areas. It is a cultivated land and grass-dominant upland watershed, with an area of approximately 3442 km^2^. In addition, the source watershed of the Ju-He River, a tributary of the Bei-Luo-He River, was also estimated, and the watershed located above Huang-Ling Hydrological Gauge Station was used as the other study area, which is a woodland-dominant upland watershed area, with an area of approximately 2274 km^2^. Both of the study areas were typical upland watersheds in the Loess Plateau, with similar elevation and slope. The average elevations of the Wu-Qi and Huang-Ling watersheds were 1544 and 1291 m, while the average slopes of the Wu-Qi and Huang-Ling watersheds were 14.7 degrees and 13.8 degrees, respectively.

The sources of original data used in this study are summarized in Table 1. Arc Hydro Tools 2.0 was employed to generate the general watershed attributes of the river line and sub-watershed boundaries based on a spatial analysis of 30 m Digital Elevation Model (DEM) maps. The 30 m grid maps of land use cover in 2015 were employed and analyzed by ArcGIS to calculate the areas of each land use type, which were classified according to China Current Land Use Classification (GB/T 21010-2007) into 25 sub-classifications, but only 14 types occurred in these two study areas. The watershed streamflows were calculated based on observed monthly river flow records divided by watershed area. These data were all used as model inputs and/or observed data in calibration or verification processes to support model applications.

### 2.2. Application of the GWLF

The Generalized Watershed Loading Functions (GWLF) model was employed to model watershed transport processes concerning streamflow and sediment yield estimations [21]. GWLF is considered to be a combined distributed and lumped parameter watershed model with reliable monthly estimations of streamflow and sediment yields as model results. For surface runoff estimations, GWLF is distributed in the sense that it divides multiple homogenous land use areas based on the Soil Conservation Service Curve Number (SCS-CN) method. For subsurface groundflow estimations, the model acts as a lumped parameter model with a linear groundwater reservoir to describe the shallow saturated subsurface zone and calculate flow yields. A daily water balance approach is operated to produce the daily hydrological process estimation and Universal Soil Loss Equation (USLE) method is used for sedimental process estimation. Regional Nutrient Management (ReNuMa) software, which uses the same model components as GWLF, was used in this subject [22]. Two new modules, the segment function approach for the saturated zone and the leakage transport approach for the unsaturated zone based on a previous study, were added to refine the model capacity during low flow periods [23,24].

The monthly records of streamflow and sediment yields from two hydrological stations (Huang-Ling and Wu-Qi, shown in Figure 1) were used to determine the parameters of the GWLF model. The observed records from 2006 to 2011 were used in the calibration process, while the records from 2012 to 2015 were reserved for model verification. Only May to October had records of monthly sediment yields, while the records of monthly streamflow were available for all the twelve months. The daily records of temperature and precipitation from the two weather stations in/around the study area were used as the input data for GWLF, while the available records from 2009 to 2015 of the precipitation stations located in the study area were used to replace the data of weather stations for better estimations. The Thiessen polygon method was used to spatially integrate the records from multiple precipitation stations for one group of daily precipitation within each studied watershed [25].

The Solver macro add-in procedure embedded in Microsoft Excel/ReNuMa, based on the nonlinear least square method, was used for model calibration based on minimizing the sum of the squared errors between the observed and estimated values. For each studied watershed, a sensitivity analysis was firstly operated to determine the sensitive parameters. Then, the hydrological process was analyzed based on observed monthly streamflow to calibrate the sensitive hydrological parameters. The sensitive sediment parameters were calibrated based on observed monthly sediment yields in sequence. The Nash–Sutcliffe coefficient ($R_{NS}^{2}$) was used for measuring model accuracy, which can be formulated as

(1) $$R_{NS}^{2}=1-\frac{\sum_{i=1}^{n}{\left(O_{i}-P_{i}\right)}^{2}}{\sum_{i=1}^{n}{\left(O_{i}-\bar{O}\right)}^{2}}$$

where $O_{i}$ indicated the observed value in month *i*, $P_{i}$ indicated the predicted value in month *i*, $\bar{O}$ indicated the mean value of observed values in all months, and n indicated the number of months being estimated. The $R_{NS}^{2}$ could take on values between −∞ and +1, with zero indicating that the model prediction is no better than the mean of the observations and +1 indicating perfect agreement. In addition, the coefficient of determination (r^2^) was also employed as the test statistic in model calibration and verification to characterize the goodness-of-fit of monthly model predictions versus observed data. The r^2^ could indicate the degree of agreement of a linear relationship between predictions and observations, with a value between 0 and +1. The detailed results of GWLF are summarized in Section 3.1.

### 2.3. Application of the LARS-WG

The Long Ashton Research Station Weather Generator (LARS-WG) was used for future weather data estimations. The LARS-WG is a stochastic weather generator based on a statistical downscaling technique [26]. It uses a series of semi-empirical distributions to describe site-scale climatic factors, and represents current and projected future climate status with a moderate data requirement and cost effective computational demand [27]. The LARS-WG can achieve a similar performance to other sophisticated models for site-scale temperature and precipitation estimations [28,29,30]. It is able to generate long synthetic series of daily weather data under various climate change scenarios, and can be adopted with confidence for climate change impact studies together with the watershed model for hydrological and sedimental estimations [31,32].

In this study, two weather stations in/around the study areas were selected to determine the local parameter set of the LARS-WG, which included Luo-Chuan (No. 53942; altitude of 1159.8 m; 109.50 °E, 35.82 °N) and Wu-Qi (No. 53738; altitude of 1331.4 m; 108.17 °E, 36.92 °N), shown in Figure 1. Sixty years of observed daily weather data for each station from 1957 to 2016 were used for parameter calibration of the LARS-WG model. Sixty years of synthetic daily weather data were generated based on the calibrated parameter set and compared with the observed data for model validation, the results of which are listed in Section 3.2.

There are four future climate change scenarios, namely, RCP2.6, RCP4.5, RCP6.0 and RCP8.5, considered in this study. They were proposed by the IPCC in their Fifth Assessment Report (AR5) and are widely used in recent climate change impact studies. Different RCP scenarios are named according to radiative forcing levels for 2100 that also have different emission and greenhouse gas concentration trajectories. RCP2.6 represents a mitigation scenario with greenhouse gas concentration peaking between 2030 and 2040 and then declining thereafter. RCP8.5 represents a very high emission scenario with continuous rising of greenhouse gas concentration throughout the 21st century. RCP4.5 and RCP6.0 are two medium stabilization scenarios. Emissions of greenhouse gas in RCP 4.5 peak around 2040 and then decline, and emissions of greenhouse gas in RCP 6.0 peak around 2080 and then decline. Various GCMs have been achieved based on different RCP scenarios to represent future climate status.

Two future periods—2041–2060 (2050s) and 2061–2080 (2070s)—were considered in this study, and the WorldClim version 1.4 dataset was adopted to obtain confident GCM outputs. The original GCM outputs had been calibrated (bias corrected) and spatially downscaled using WorldClim 1.4 and released as refined global raster data with various resolutions [33]. There are a number of different GCMs, and they give different results. All the eleven available GCM outputs in WorldClim were considered, i.e., those which had complete results for four RCP scenarios and two future periods. An ensemble means based on the averages of eleven different GCM outputs was achieved for future climatic factor estimations to avoid uncertainty. The GCMs used in this study are summarized in Table 2.

The global raster data at a 2.5 min spatial resolution in WorldClim 1.4 are adopted. They use one raster to represent 2.5 min of a longitude/latitude degree, which is approximately 4.5 km at the equator. The raster maps were estimated by Spatial Analyst Tools in ArcGIS 10.2, with batch commands in Python to estimate the changes in monthly precipitation and mean daily maximum and minimum temperature in the 2050s and 2070s under four RCP scenarios for each GCM. The average changes in the eleven GCMs were used to build the scenario analysis files of the LARS-WG, based on which, the model parameters were updated and used to generate future site-scale daily weather data [34]. Sixty years of synthetic daily weather data for each station were generated towards four RCP scenarios in two future periods to represent the predicted future climate conditions. These synthetic weather data were further used as input data for the GWLF model to estimate the responses of the watershed hydrological and sedimental processes, which are discussed in detail in Section 4.

## 3. Results

### 3.1. GWLF Model

**Figure 2:**
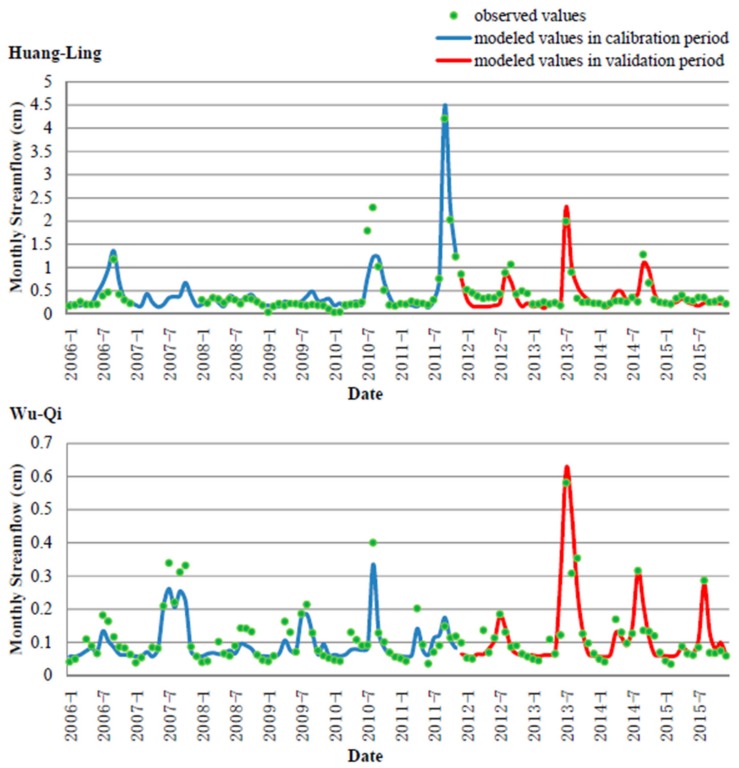
Time series of observed and modeled monthly streamflow in the two studied watersheds.

The observed monthly streamflow records for each studied watershed were firstly used to calibrate the transport parameters of the GWLF model, which mainly included Curve Number (CN) values for each land use type, evaporation cover factor for each month, two-level recession coefficient and seepage coefficient, and unsaturated leakage coefficient. The calibrated parameter set could describe the watershed hydrological process, based on which, the monthly streamflows were modeled and compared with the observed records to estimate the model performance (Figure 2). The results showed that both of the model applications in the two studied watersheds had reasonable accuracy. For the Huang-Ling watershed, the statistic of $R_{NS}^{2}$ was 0.87 and the statistic of r^2^ was 0.88 during the calibration period (2006–2011), while the $R_{NS}^{2}$ was 0.77 and the r^2^ was 0.83 during the verification years (2012–2015). We found that both the statistics slightly declined in the verification period, but all the values of $R_{NS}^{2}$ and r^2^ were higher than 0.75, which was in accordance with the general accuracy level of watershed model applications for monthly hydrological process estimation. Similarly, the $R_{NS}^{2}$ and the r^2^ for the Wu-Qi watershed were 0.81 and 0.88 during the calibration period and 0.76 and 0.82 during the verification period, with no values below 0.75. These results indicated that the calibrated GWLF model could afford reliable modeled results of streamflow, based on which, the calibration of sediment parameters could be performed.

**Figure 3:**
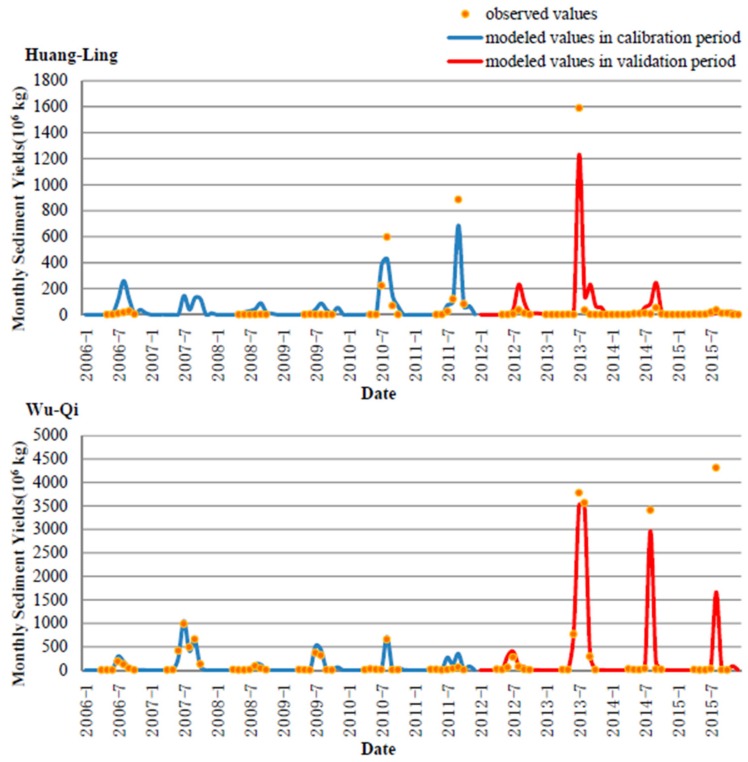
Time series of observed and modeled monthly sediment in the two studied watersheds.

The calibrated sedimental parameters mainly included the USLE parameter for each land use, erosive coefficient for each month, power of sediment transport capacity, and sediment delivery ratio. Time series of observed and modeled monthly sediment yields during the study period are shown in Figure 3. We found that during the calibration period, the values of $R_{NS}^{2}$ and r^2^ were 0.87 and 0.90 for the Wu-Qi watershed, and were 0.85 and 0.87 during the verification period with a slight decline similar to the streamflow process. There was a great error for the simulation of monthly sediment in July 2015 as the observed value was much larger than the simulated peak. However, the model outputs could fit well with several other peaks, and the test values were acceptable. Therefore, we could treat the sole underestimation as an outlier and accept the model accuracy. For the Huang-Ling watershed, the values of $R_{NS}^{2}$ and r^2^ were 0.81 and 0.84 during the calibration period, and were 0.88 and 0.91 during the verification period, with even a slight rise. The better test values in the verification period mainly resulted from the great goodness-of-fit for the high sediment yields that occurred in June 2013.

These results showed that the calibrated GWLF model had great abilities in modeling the watershed hydrological and sedimental processes of the study areas, based on which, the response estimations towards future climate changes could be implemented with the linkages of the LARS-WG model results.

### 3.2. The LARS-WG Model

The parameters set in the LARS-WG model for the two studied weather stations, Luo-Chuan and Wu-Qi, were calibrated based on sixty years of daily weather records, respectively. The alternative random seed number of 541 was used to generate synthetic daily weather for Luo-Chuan, and the random seed number of 1987 was used for Wu-Qi. Sixty years of synthetic daily weather data for each site were generated and compared with the observed daily weather data to assess the ability of the LARS-WG model following routine model applications based on three general statistical tests, which included the Kolmogorov–Smirnov (K–S) test, the *t*-test, and the *F*-test. The ratios of significantly different results at the 5% significance level out of the total number of tests are summarized in Table 3—a small number of which indicated a good model performance.

Here, eight weather items were estimated, including seasonal wet/dry series distribution (WDSeries), daily precipitation distribution (PrecD), daily minimum temperature distribution (TminD), daily maximum temperature distribution (TmaxD), monthly mean of precipitation (PMM), monthly mean of daily maximum temperature (TmaxM), monthly mean of daily minimum temperature (TminM), and monthly variances of precipitation (PMVs). We found that most weather items had no significant differences between the observed and modeled datasets, indicating a high modeling precision in this instance. As a common issue among statistical downscaling models with a wet/dry-series based framework [35], the PMVs in the twelve tests were significantly different for each of the two study cases, which occurred in March and April. This issue indicated a relatively weak modeling ability of the LARS-WG for monthly precipitation variability in spring. In addition, there was one significantly different result occurring in August for TminM estimation in Luo-Chuan, and one in autumn for dry series distribution estimation in Wu-Qi. However, the results were acceptable overall within the same level of other LARS-WG model applications [36,37] and could qualify for supporting further scenario analysis.

Future weather data representing changed climate status were generated through updating the calibrated parameter set of the LARS-WG model based on the integrated GCM outputs. The scenarios of RCP2.6, RCP4.5, RCP6.0 and RCP8.5 during the periods of 2046~2065 (Period A, simply as PA below) and 2080~2099 (Period B, simply as PB below) were estimated. Sixty years of daily precipitation and daily Tmax and Tmin were generated for each scenario and period, which were further used as the input data for the calibrated GWLF to further estimate watershed hydrological and sedimental processes. All parameters used in GWLF and LARS-WG can be found in the Supplementary Materials. Various responses to different climate change scenarios in different landscapes are compared and discussed in detail in Section 4.

## 4. Discussion

### 4.1. Comparison of Responses in Hydrological Processes

**Figure 4:**
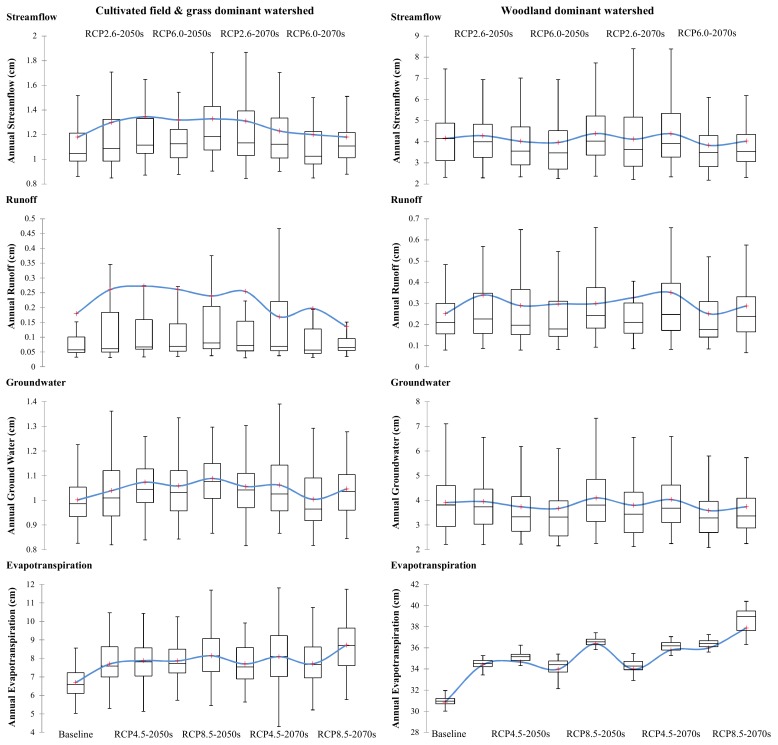
The changes in annual streamflow, runoff, groundwater and evapotranspiration in response to various future climate changes in the 2050s and 2070s. The upper and lower borders of the box represent the 25th and 75th percentiles; the line and cross in the box interior represent the median and mean values; the “whiskers” represent the lower and upper limits.

The current and future average annual streamflow, as well as runoff, groundwater, and evapotranspiration, in the cultivated field and grass-dominant watershed of Wu-Qi (called CGW for short below) and the woodland-dominant watershed of Huang-Ling (called WW for short below) are illustrated in Figure 4. A series of box plots were used to represent the distribution characteristics of each sample. Sixty years of GWLF results for each scenario group were used to obtain statistics to draw these box plots with the Statistical Software for Excel (STATXL). The length of the box represents the distance between the 1st quartile (25th percentile) and 3rd quartile (75th percentile), and the line and cross in the box interior represent the median and mean values in the group, respectively. The ends of the “whiskers” are the lower and upper limits, the values outside of which were considered as anomalous.

The streamflow yields in WW were higher than those in CGM, indicating a better water conservation capacity of woodland than cultivated field in the study area. From the point of view of average annual yields per unit area, the streamflow in WW was approximately 3.5 times that of the streamflow in CGM for the baseline status, while the average annual precipitation in WW was only approximately 1.4 times that of the precipitation in CGM. The streamflow was composed of runoff and groundwater. On one hand, the runoff in WW was slightly higher than that in CGM, but generally of the same order of magnitude. The variation in runoff in CGM was greater than that in WW. For each group of annual runoff samples under the same period and weather conditions, the mean values in CGM were higher than the median values, while the mean and median values in WW were always on the same level. This meant that there were more extreme events with high runoff yields in CGM, which indicated a poor capacity of cultivated field as a buffer facing intense rainfall. On the other hand, there was obviously more groundwater yield in WW than in CGM. The groundwater flow in WW was nearly four times that of the groundwater in CGM, which accounted for the main source of more streamflow in WW than CGM. The woodland underlying surface was of benefit for rainwater infiltration to recharge the unsaturated zone, and the saturated zone had better water storage capacity for less water losses from leakage and/or seepage processes. However, the great rainwater blocking capacity of woodland also provided more available moisture in soil for evapotranspiration. The evapotranspiration in WW was more than 4.5 times that of the evapotranspiration in CGM. The differences in potential evapotranspiration capacity between CGM and WW are one of the most important factors leading to different responses of streamflow to different hotter and wetter climate change scenarios.

Based on the model outputs for various climate change scenarios during two future periods of the 2050s and 2070s, the amount of annual streamflow in CGM would generally increase and go through a process of increasing firstly and then decreasing. However, there was no consistent increasing trend for the amount of annual streamflow in WW, with several decreases under scenarios of high radiative forcing levels. In CGM, the streamflow would increase in the 2050s for all scenarios, among which RCP4.5 and RCP8.5 would increase more. In the 2070s, the streamflow would decrease compared to the values in the 2050s for all scenarios except RCP 2.6, which was still at the level of the 2050s. There were significantly negative effects on the streamflow under high radiative forcing conditions in the 2070s, i.e., the higher the radiative forcing levels, the less the streamflow. For the scenario of RCP 8.5 in the 2070s, the streamflow would return to the baseline level even if the precipitation increased by 15.1%, which was offset by the intense increase in evapotranspiration due to higher temperature. In WW, the streamflow in the 2050s would decrease under the medium radiative forcing levels of RCP 4.5 and RCP 6.0, but slightly increase for the scenarios of RCP 2.6 and RCP 8.5. In the 2070s, the streamflow would decrease except RCP 4.5, which increased by 5.2% relative to the baseline level. The decrease in streamflow under RCP 6.0 was most significant, decreasing by 4.7% in the 2050s and 7.9% in the 2070s. The negative effect on streamflow under RCP 2.6 was the lowest, with an increase of 3.0% in the 2050s and a decrease of 0.9% in the 2070s.

In another view, the amount of annual runoff in both CGM and WW would go through a process of increasing firstly in the 2050s and then decreasing in the 2070s. In the 2050s, there were increasing trends of runoff for both CGM and WW, with a negative correlation with radiative forcing levels, i.e., the higher the radiative forcing level, the less the increase in runoff. In the 2070s, runoff in both CGM and WW represented decreasing trends under high radiative forcing levels. In CGM, the amount of annual runoff would decrease and return back to the baseline level for the scenarios of RCP 4.5, RCP 6.0 and RCP 8.5. In WW, only the scenarios of RCP 6.0 and RCP 8.5 would result in decreases in runoff yields, indicating a better resistance stability of WW to offset the impact of climate change with high radiative forcing levels on runoff. The amount of annual groundwater flow in CGM would generally increase, with a trend of increasing at first and then decreasing like the streamflow. However, the amount of annual groundwater flow in WW would suffer continuous decreases, with greater degrees under high radiative forcing levels due to more evapotranspiration. Changes in the hydrological process would alter the original mechanisms of sediment delivery for erosion in CGM and WW, which are discussed in the next section.

### 4.2. Comparison of Responses on Sedimental Processes

**Figure 5:**
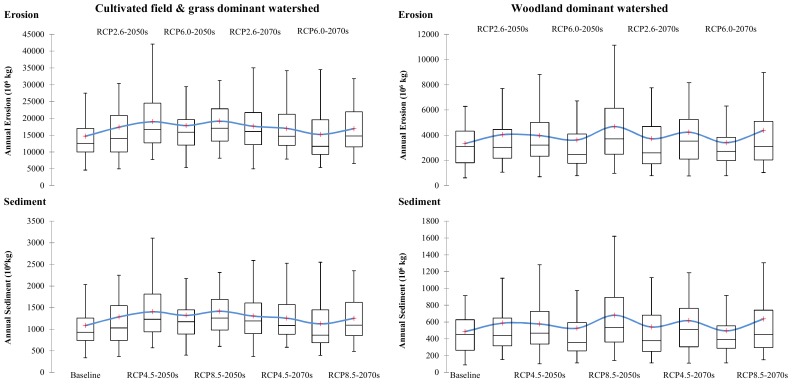
The changes in annual erosion and sediment in response to various future climate changes in the 2050s and 2070s. The upper and lower borders of the box represent the 25th and 75th percentiles; the line and cross in the box interior represent the median and mean values; the “whiskers” represent the lower and upper limits.

**Figure 6:**
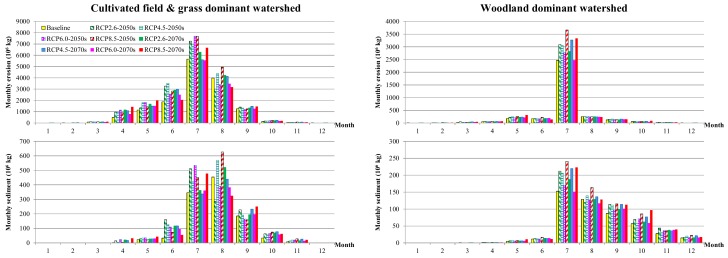
The changes in monthly erosion and sediment in response to various future climate changes in the 2050s and 2070s.

The current and future average annual erosion and sediment in CGM and WW are illustrated in Figure 5 with a series of box plots based on sixty years of data for each group. In addition, the average monthly erosion and sediment in CGM and WW for baseline and two future periods under various climate change scenarios are also summarized in Figure 6.

The results of the model scenario analysis showed that there were significantly more erosion and sediment yield in CGM than WW, indicating better soil and water conservation capacities of the woodland surface. The erosion was directly created by rainfall with different intensities in different land use areas. It can be seen as a source reservoir of sediment available to be delivered. A part of erosion was transferred by runoff to generate the sediment yield in the water body. The amount of annual erosion yields per unit area in CGM was 4.4 times that of the erosion in WW for the baseline status, implying poorer soil-fixing capacity in the cultivated field. However, the average annual sediment in CGM was only 2.2 times that of the sediment in WW, implying a higher delivery ratio of erosion in woodland. The greater sediment transport capacity of woodland mainly resulted from its higher runoff yields.

In another view of monthly yield estimations, the erosion in CGM mainly occurred from April to September, with the most in July. The erosion in WW mainly focused on July, with small amounts in May, June, September and October. The erosion in July accounted for 73.8% of the year in WW, but only 38.6% in CGM. However, the amount of erosion in CGM in July was still 2.3 times that of WW. The distributions of erosion were generally consistent with the distribution of precipitation, but different in terms of amounts between CGM and WW in spring and autumn. The erosion of WW was significantly lower than that of CGM in months with mild precipitation, indicating stronger soil-fixing capacity of WW to avoid erosion occurring in non-extreme precipitation conditions. The sediment in CGM mainly occurred from June to Autumn, with small amounts in May, June and October. The erosion in WW mainly occurred in the second half of the year, with the highest yields in July and then monthly declines.

Focusing on potential changes in the future, both CGM and WW had higher erosion and sediment yields under all climate change scenarios. There were generally more increases in annual erosion and sediment in the 2050s. The greatest increases in annual erosion and sediment in WW were found for the scenario of RCP8.5 in the 2050s. In CGM, the increases in annual erosion and sediment were significant for the scenarios of RCP4.5 and RCP8.5 in the 2050s. In the 2070s, there was generally less annual erosion and sediment than the 2050s under the same RCP scenarios in CGM. However, annual erosions and sediments in the 2070s were still at a high level in WW, and an increasing trend could be found under the scenario of RCP4.5. Both CGM and WW were not sensitive to the RCP6.0 scenario, under which the annual erosions and sediments in the 2070s were all similar to the baseline level.

For the two future periods under various climate change scenarios, there were general increases in monthly erosion and sediment yields, with several exceptions in hot summer months. In CGM, the declines in monthly erosion and sediment mainly occurred in August and September. There were significant decreases in monthly erosion in August under the scenarios of RCP2.6 and RCP6.0 in the 2050s, and RCP 6.0 and RCP 8.5 in the 2070s. In September, the decreases in monthly erosion could be found under the scenarios of RCP6.0 and RCP8.5 in the 2050s. Similar features could also be found in the changes in monthly sediment in CGM. In WW, there were only two significant decreases in monthly erosion, occurring in August under the scenario of RCP2.6 in the 2050s and RCP 8.5 in 2070s. However, the changes in monthly sediment in August in WW were generally negative, indicating a weaker capacity for sediment delivery of woodland in August under the future climate change scenarios.

To sum up, future climate changes could lead to increases in erosion and sediment in both CGM and WW. Although the relative increasing percentage was higher, the absolute increasing amount of erosion and sediment in WW was significantly lower than CGM. The peak of increase appeared in the near future period of the 2050s, indicating great demands for rapid response in management. For sediment control towards future climate change status, various policies to expand the area of woodland, such as returning farmland to forests and afforestation, should be primarily encouraged. Besides, integrated soil conservation measures in cultivated land needed to be implemented to reduce erosion yields. In addition, suitable facilities, such as vegetable buffers and artificial wetlands, could be built to block the transfers of erosion for lower sediment delivery ratios.

## 5. Conclusions

This work estimated the responses of watershed hydrological and sedimental processes to future climate change in two typical watersheds in the Chinese Loess Plateau with different dominant land use covers of woodland and cultivated field, respectively. The following three main conclusions can be drawn from the study:

(1) There was a hotter and wetter trend of climate change in the study area. The amount of streamflow in CGM would increase firstly and then decrease, with the peak level appearing in the 2050s under the scenario of RCP 4.5. The changes in streamflow in WW were generally negative due to intense evapotranspiration in hot weather conditions, which could offset the increased precipitation. The runoff in both CGM and WW would increase in the 2050s, which is conducive to sediment delivery.
(2) There were increasing trends of erosion and sediment yields in both CGM and WW under all climate change scenarios. The woodland represented greater resistance stability than cultivated field, with a lesser increment in sediment in hotter and wetter weather conditions. The sediment yields in both CGM and WW would reach their peak in the 2050s in the near future, indicating urgent demands for sediment control.
(3) There is great demand for effective management measures to address the impacts of future climate change on sediment yields. The strategy of increasing woodland areas is one of the most effective approaches, such as through policies of afforestation and returning farmland to forests. Without essential land use conversion, various soil conservation measures should be implemented in cultivated land to reduce erosion yields, including conservation tillage, cover crops, crop rotations, contour farming and terraces, etc. In addition, a series of installations that could block the delivery of sediment from erosion should be constructed, such as vegetable buffers, man-made drainage courses and wetlands.
(4) The approach of integrated multi-model applications used in this study showed great ability to quantitatively estimate the responses of hydrological and sedimental processes to future climate change in the Yellow River Basin and may also be applicable in other areas with similar conditions.

## Figures and Tables

**Table 1:** Sources of the input data used in this study.

Name	Source	Resolution	Remark
Digital Elevation Model	Geospatial Data Cloud site, Computer Network Information Center, Chinese Academy of Sciences. (http://www.gscloud.cn)	30 m × 30 m raster	Advanced Spaceborne Thermal Emission and Reflection Radiometer Global Digital Elevation Model Version 2 (ASTER GDEM V2)
Land Use Cover Maps	Data Center for Resources and Environmental Sciences, Chinese Academy of Sciences (RESDC) (http://www.resdc.cn)	30 m × 30 m raster	2015
Weather Data	Climatic Data Center, National Meteorological Information Center, China Meteorological Administration (http://data.cma.cn)	Daily temperature and precipitation	1957–2016
River Flow Records	Annual Hydrological Report P.R. China (National Library of China)	Monthly mean rate of flow	2006–2015
Sediment Records	National Earth System Science Data Sharing Infrastructure, National Science and Technology Infrastructure of China (http://www.geodata.cn)	Monthly mean sediment concentration in flow	2006–2015

**Table 2:** Summary of global climate models used in this study.

Abbreviation	Full Name
BCC-CSM1-1	Beijing Climate Center Climate System Model version 1.1
CCSM4	Community Climate System Model Version 4
GISS-E2-R	Russell Ocean Model of NASA Goddard Institute for Space Studies
HadGEM2-AO	Hadley Centre Global Environmental Model version 2, Atmosphere-Ocean
HadGEM2-ES	Hadley Centre Global Environmental Model version 2, Earth System
IPSL-CM5A-LR	The Low-Resolution Version of Institut Pierre Simon Laplace Earth System Model for the Coupled Model Project Phase 5
MIROC-ESM-CHEM	Atmospheric Chemistry Coupled Earth System Model of Model for Interdisciplinary Research on Climate
MIROC-ESM	Earth System Model of Model for Interdisciplinary Research on Climate
MIROC5	Model for Interdisciplinary Research on Climate Version Five
MRI-CGCM3	Meteorological Research Institute Coupled Global Climate Model Version 3
NorESM1-M	The Norwegian Climate Center’s Earth System Model

**Table 3:** Results of the statistical tests comparing the observed data and synthetic data generated by the Long Ashton Research Station Weather Generator (LARS-WG) with the numbers of tests revealing significantly different results at the 5% significance level.

Items	Total Tests	Number of Significant Differences		Percentage of Significant Differences (%)	
		Luo-Chuan	Wu-Qi	Luo-Chuan	Wu-Qi
WDSeries ^a^	8	0	1	0	12.5
PrecD ^b^	12	0	0	0	0
PMM ^c^	12	0	0	0	0
PMV ^d^	12	1	1	8.3	8.3
TminD ^e^	12	0	0	0	0
TminM ^f^	12	1	0	8.3	0
TmaxD ^g^	12	0	0	0	0
TmaxM ^h^	12	0	0	0	0

^a^: indicates seasonal wet/dry series distributions tested by the Kolmogorov–Smirnov (K–S) test. ^b^: indicates daily precipitation distributions tested by the Kolmogorov–Smirnov (K–S) test. ^c^: indicates monthly mean of precipitation tested by the *t*-test. ^d^: indicates monthly variances of precipitation tested by the *F*-test. ^e^: indicates daily minimum temperature distributions tested by the Kolmogorov–Smirnov (K–S) test. ^f^: indicates monthly mean of daily minimum temperature tested by the *t*-test. ^g^: indicates daily maximum temperature distributions tested by the Kolmogorov–Smirnov (K–S) test. ^h^: indicates monthly mean of daily maximum temperature tested by the *t*-test.

## Supplementary Materials

The following are available online at https://www.mdpi.com/1660-4601/16/20/4054/s1.
