# Supplementary material for: Estimating the Responses of Hydrological and Sedimental Processes to Future Climate Change in Watersheds with Different Landscapes in the Yellow River Basin, China
Source: Int J Environ Res Public Health. 2019 Oct 22;16(20):4054. doi: 10.3390/ijerph16204054 (PMC6843980; doi:10.3390/ijerph16204054)
Supplement: Supplementary file 1 [file ijerph-16-04054-s001.zip › Supplementary materials/Parameters of GWLF.docx]

**Table S1.** Calibrated results of GWLF hydrological transport parameters and sedimental parameters.

| **Parameter Items** | **Subcategories** | Wu-Qi | Huang-Ling |
| --- | --- | --- | --- |
| runoff curve number | Cultivated land | 47.04 | 47.04 |
|  | Wood land | 21.38 | 21.38 |
|  | Shrubbery lands | 30.47 | 31.25 |
|  | Sparsely forested woodland | 24.60 | 24.60 |
|  | Other forest land including garden | 35.06 | 25.06 |
|  | High coverage grassland | 41.56 | 32.65 |
|  | Middle coverage grassland | 51.40 | 41.04 |
|  | Low coverage grassland | 57.84 | 57.84 |
|  | Reservoir and pond | 100.0 | 100.0 |
|  | Bottomland | 98.37 | 98.37 |
|  | Other Unused lands | 88.67 | 88.67 |
|  | Cities and towns | 84.83 | 84.83 |
|  | Rural residential land | 74.66 | 74.66 |
|  | Other developed land | 79.66 | 79.66 |
| et cover factor | JAN | 0.1 | 0.1 |
|  | FEB | 0.1 | 0.1 |
|  | MAR | 0.1 | 0.1 |
|  | APR | 0.6 | 0.6 |
|  | MAY | 0.6 | 0.6 |
|  | JUNE | 0.6 | 0.6 |
|  | JULY | 0.6 | 0.6 |
|  | AUG | 0.6 | 0.6 |
|  | SEPT | 0.6 | 0.6 |
|  | OCT | 0.1 | 0.1 |
|  | NOV | 0.1 | 0.1 |
|  | DEC | 0.1 | 0.1 |
| Groundwater flow | Recession coefficient | 0.0008 | 0.0034 |
|  | Seepage coefficient | 0.0466 | 0.0245 |
|  | Slow recession coefficient | 0.0007 | 0.0131 |
|  | Slow seepage coefficient | 0.0001 | 0.0245 |
|  | Ground Water Limit for recession | 3.0000 | 1.0000 |
|  | Ground Water Limit for seepage | 3.0000 | 1.0000 |
|  | Unsaturated zone leakage coefficient | 0.3453 | 0.2930 |
| USLE parameter | Cultivated land | 0.2134 | 0.2134 |
|  | Wood land | 0.0010 | 0.0010 |
|  | Shrubbery lands | 0.0050 | 0.0050 |
|  | Sparsely forested woodland | 0.0100 | 0.0100 |
|  | Other forest land including garden | 0.0080 | 0.0080 |
|  | High coverage grassland | 0.0160 | 0.0160 |
|  | Middle coverage grassland | 0.0199 | 0.0199 |
|  | Low coverage grassland | 0.0498 | 0.0498 |
|  | Reservoir and pond | 0.0000 | 0.0000 |
|  | Bottomland | 0.0000 | 0.0000 |
|  | Other Unused lands | 0.0100 | 0.0100 |
| Erosivity coefficient | JAN | 0.18 | 0.18 |
|  | FEB | 0.18 | 0.18 |
|  | MAR | 0.18 | 0.18 |
|  | APR | 0.68 | 0.18 |
|  | MAY | 0.68 | 0.42 |
|  | JUNE | 0.68 | 0.18 |
|  | JULY | 0.88 | 1.57 |
|  | AUG | 0.58 | 0.18 |
|  | SEPT | 0.48 | 0.18 |
|  | OCT | 0.18 | 0.18 |
|  | NOV | 0.18 | 0.18 |
|  | DEC | 0.18 | 0.18 |
| Sediment delivery ratio | | 0.0739 | 0.1455 |
